# Predicting the Bending of 3D Printed Hyperelastic Polymer Components

**DOI:** 10.3390/polym15020368

**Published:** 2023-01-10

**Authors:** Lucas Gallup, Mohamed Trabia, Brendan O’Toole, Youssef Fahmy

**Affiliations:** Department of Mechanical Engineering, University of Nevada, Las Vegas, NV 89154, USA

**Keywords:** 3D printing, hyperelastic, thermoplastic polyurethane, bending deflection, NinjaFlex^®^

## Abstract

The advancement of 3D printing has led to its widespread use. NinjaFlex^®^, a thermoplastic polyurethane (TPU) filament, is a highly durable and flexible material that has been used to create flexible parts. While this material has been available for nearly two decades, the mechanical properties of 3D printed NinjaFlex^®^ parts are not well-understood, especially in bending. The focus of this research was predicting the behavior of small 3D printed NinjaFlex^®^ components. Three-dimensionally printed rectangular specimens of varying lengths and aspect ratios were loaded as cantilevers. The deflection of these specimens was measured using a computer. The experimental results were compared to a modified form of the Euler–Bernoulli Beam Theorem (MEB), which was developed to account for nonlinearities associated with large deflection. Additionally, experimental results were compared to the finite element analysis (FEA). The results showed that both modeling approaches were overall accurate, with the average difference between experimental deflection data and MEB predictions ranging from 0.6% to 3.0%, while the FEA predictions ranged from 0.4% to 2.4%. In the case of the most flexible specimens, MEB underestimated the experimental results, while FEA led to higher retraction.

## 1. Introduction

Scientific and technological advancements have allowed using polymer synthesis to develop materials for many applications. An example of these new materials is the hyperelastic thermoplastic polyurethanes (HTPUs). HTPUs offer the manufacturing advantage of being processed as a thermoplastic while maintaining properties similar to rubber due to their molecular structure, which contains two specific regions: soft and hard. The soft regions allow for high flexibility, while the hard ones contribute to permanent deformation, tensile strength, and hysteresis of the material [1,2]. This combination provides HTPUs with a composite-like behavior that is hyperelastic and non-linear [2].

Simultaneously, hardware development has allowed 3D printing to speed up the process of developing prototypes and part production, especially when a relatively small numbers of components are needed. However, incorporating 3D printing in the industry has been limited by changes that the materials, especially polymers, experience during material deposition. These changes affect the mechanical behavior as compared to traditionally manufactured materials because 3D printing introduces a plethora of factors including print orientation, infill raster angle, infill density, and shell thickness. In the following, a brief overview of research in this area is presented.

Some mechanical properties of hyperelastic polymers can be readily determined using uniaxial tensile testing. A brief overview follows of relevant research in this area. By 3D printing dog-bone shaped specimens from Elastosil M4061 and translucent soft silicones, stress–strain curves as well as elastic moduli were obtained [3]. The effects of infill density on uniaxial tensile strength of 3D printed Polylactic acid (PLA) specimens were considered [4]. Increasing the infill density consistently increased the modulus of elasticity, yield strength, and ultimate strength. The variation in the mechanical characteristics of Polyamide 12 (PA12 or Nylon 12) was studied with respect to changing the laser power and hatch orientation; it was found that 95% full power at 0° with respect to the printer’s *x*-axis resulted in higher tensile strength, modulus of elasticity, and elongation at break [5]. The effects of varying the printer platform temperature and print speed were studied for Onyx, a Nylon filament mixed with carbon fibers [6]. It was found that increasing either printer platform temperature or print speed caused an increase in modulus of elasticity and tensile strength. The effects on tensile properties of polyetherimide (PEI) caused by print speed and nozzle temperature were studied, and it was found that increasing either parameter caused a decrease in modulus of elasticity [7].

The influence of infill raster angle on various polymers including NinjaFlex^®^ was assessed under uniaxial tensile loading [8]. It was found that as the raster angle approached the loading direction, these materials’ moduli of elasticity increased. The ultimate tensile strength of NinjaFlex^®^ increased as the raster angle approached the loading direction. The infill density of NinjaFlex^®^ was varied, and its effects on hardness, maximum stress, and flexure force were studied [9], indicating that the hardness increased as the infill density increased. The maximum stress increased with infill density for NinjaFlex^®^. The flexural force increased until 70/80% infill density, then dropped slightly for the higher density values. The effects of infill density were evaluated on 3D printed NinjaFlex^®^ hollow cells with gyroid units of varying unit sizes while under compressive loads [10]. The resulting stress–strain curves showed that as the size of cell unit increased, the stress at any given strain decreased. Similarly, the modulus of elasticity increased as the cell size decreased. The influence of infill density and print orientation on NinjaFlex^®^ under uniaxial tensile loading was considered with respect to ultimate strength and modulus of elasticity [11]. By varying the printing orientation, it was found that ultimate stress increased when the printing orientation was parallel to the loading direction rather than perpendicular to it. The modulus of elasticity remained relatively constant for specimens with a print orientation parallel to the direction of loading when the infill density increased. On the other hand, specimens with a printing orientation perpendicular to the loading direction had the modulus of elasticity increase as the infill density increased. The effects of the shell thickness on NinjaFlex^®^ 3D printed specimens with shell thickness varying between one and four filament lines were considered [12]. Uniaxial tensile testing showed that tensile strength and modulus of elasticity increased in proportion to the number of wall lines. 

While these studies addressed important questions related to the mechanical behavior of the 3D printed polymers, the bending behavior of 3D printed HTPUs under bending causing large deformation is not well-understood. This study aimed to address this question.

The first objective of this research was to characterize the behavior of 3D printed small components made of NinjaFlex^®^. Multiple rectangular specimens of varying lengths and aspect ratios were 3D printed and loaded as cantilevers. The second objective was to determine if the experimental results could be simulated with a modified form of the Euler-Bernoulli Beam Theorem (MEB) and finite element analysis (FEA). 

## 2. Materials and Methods

### 2.1. Materials and Preparation

The manufacturer of NinjaFlex^®^ has provided several key mechanical properties that were found following ASTM D638 standards [13] (Table 1). However, other researchers have provided different values for these characteristics. This variation may be due to the 3D printer used as well as the printing parameters. Additionally, some researchers have provided different stress–strain curves for NinjaFlex^®^ [8,10,11,12,14].

**Figure 1:**
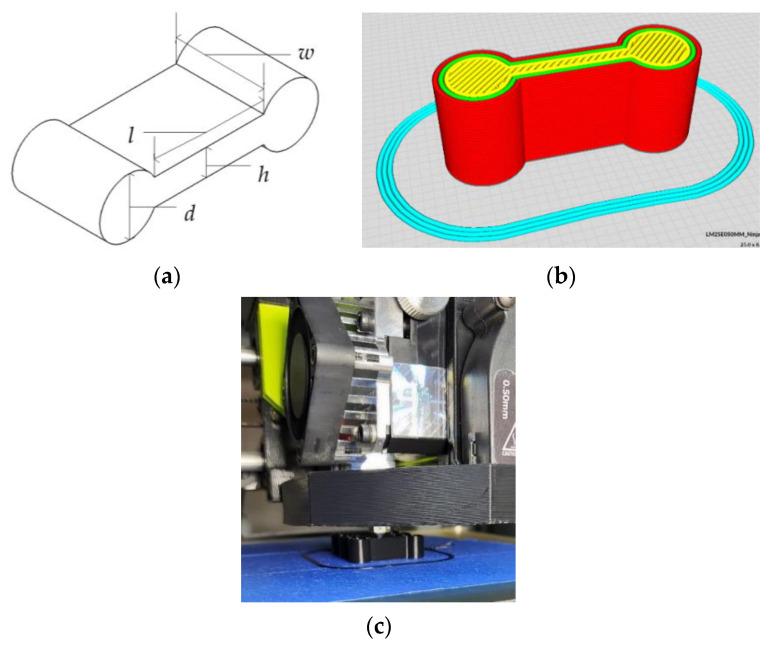
Specimen preparation: (**a**) variables of the specimens; (**b**) a specimen representation in the Cura Lulzbot^®^ Edition software; (**c**) printing a specimen using the LulzBot Taz Mini 2^®^.

In this research, experiments were conducted using 3D printed NinjaFlex^®^ dog-bone specimens with varying rectangular cross sections (Figure 1a). The length (*l*), width (*w*), and height (*h*) were combined to create eighteen unique specimens, Table A1. The diameters of the two cylindrical ends, *d*, depended upon the height of the specimen. Each of the eighteen specimens were printed three times for a total of fifty-four samples.

The CAD models of the specimens were converted to .stl files using the Cura Lulzbot^®^ Edition (model slicing software, Fargo Additive Manufacturing Equipment 3D, LLC, Fargo, ND, USA) [16] (Figure 1b)**.** This software has a library of print parameters for numerous filament materials including NinjaFlex^®^. Print quality was set to standard; print parameters are listed in Table 2. The number of wall lines for each specimen varied depending on the specimen height, *h*, as shown in Table A1. All specimens were printed on the same machine with the same black NinjaFlex^®^ filament using a Lulzbot Taz Mini 2^®^ with an Aerostruder^®^ tool head (3D printer, Fargo Additive Manufacturing Equipment 3D, LLC, Fargo, ND, USA) [17], with an extruder diameter of 0.5 mm. A single layer of painters’ tape was used on the heated print plate for print adhesion (Figure 1c).

### 2.2. Experiment

**Figure 2:**
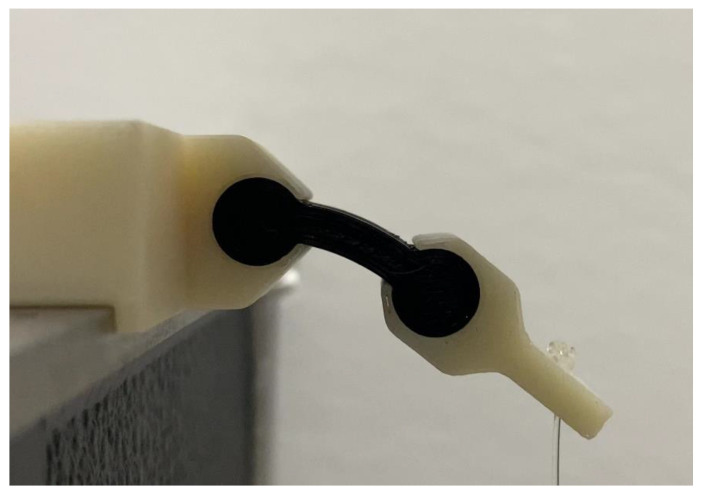
A loaded specimen between brackets.

To apply the proper boundary conditions to the two ends of the specimens, ABSplus P430 (3D printer filament, Stratasys, Ltd., Eden Prairie, MN, USA) [18] custom brackets were designed (Figure 2) and printed using a Stratasys^®^ Fortus 250mc (3D printer, Stratasys, Ltd., Eden Prairie, MN, USA) [19]. The left bracket, which was attached to a fixed base, allowed one end of the specimens to be completely fixed while the right bracket, which had a hole where weights were attached to a fishing line, bent the specimens as cantilevers. Three sets of brackets were created to tightly fit the three specimen diameters, *d*. Loads were specimen-dependent and chosen to induce large bending in the specimens (Table A2). Stiffer specimens were, therefore, subjected to heavier loads. Loads were applied in a quasi-static manner by incrementally increasing weight attached to the right bracket, as shown in Table A2. The whole experiment was recorded using an iPhone 11 Pro^®^ (cellphone, Apple Inc., Cupertino, CA, USA) at 4k resolution at a rate of 60 frames per second.

**Figure 3:**
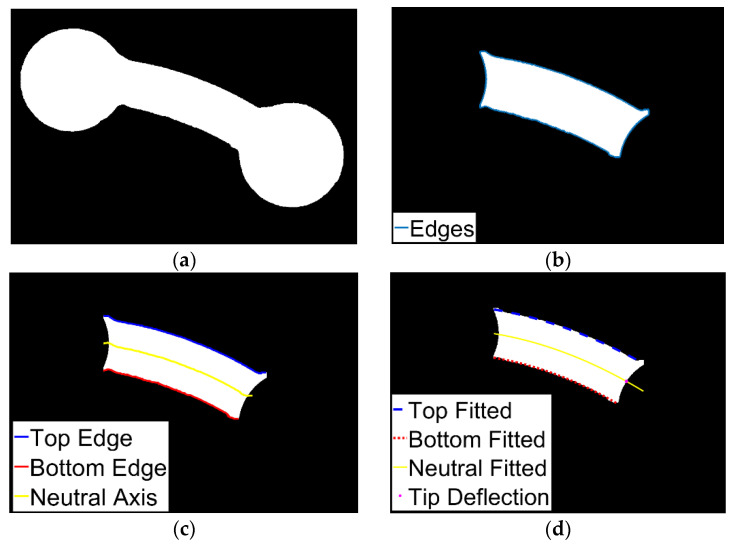
Specimen image processing: (**a**) images were cropped and binarized; (**b**) the cylindrical ends were identified and removed, and the edges of the middle portion were determined; (**c**) two sets of equally spaced points were created on the top (blue) and bottom (red) borders and averaged to determine the neutral axis (yellow); (**d**) the top and bottom edges and the neutral axis were fitted with 2^nd^ order polynomials, and the intersection of the neutral axis and the right cylindrical section was used for the tip point deflection (pink).

For each specimen, frames corresponding to a steady-state configuration after the load was applied were extracted from the videos of the experiment; corresponding loads were recorded. A custom code utilizing the MATLAB^®^ Image Processing Toolbox (software, The MathWorks, Natick, MA, USA) [20] was written for processing the images to extract the location of the tip point deflection of the free length, *l*. First, all images were cropped to reduce unnecessary noise. Next, each image was binarized to isolate the specimen (Figure 3a). The two cylindrical ends of a specimen were identified using morphological detection and removed to leave the middle section (Figure 3b). The four edges of this section were determined using a border detection function, *bwboundaries*. To identify the neutral axis specimen’s middle section, an equal number of equally spaced points were created on the top and bottom edges. For each corresponding point on these two curves, the midpoint was identified to be on the neutral axis (Figure 3c). Second-order polynomials were fitted to the top and bottom edges and the neutral axis to reduce any noise associated with the averaging (Figure 3d). The last point on the neutral axis of the rectangular section was identified by the intersection of the two cylindrical sections with the neutral axis curve (Figure 3d). Finally, all relevant information was converted from pixels to millimeters based on camera parameters.

### 2.3. NinjaFlex^®^ Material Model

**Figure 4:**
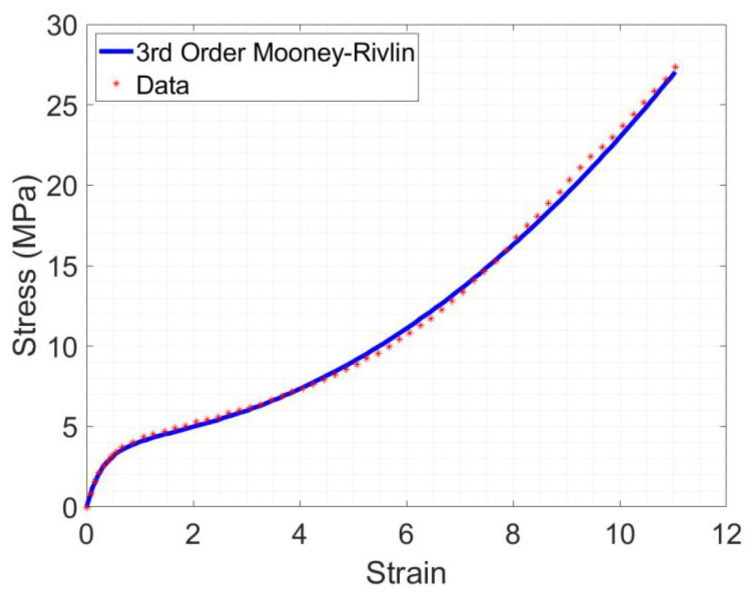
Third-order Mooney–Rivlin model fit to uniaxial tensile test data.

The material model used in the finite element analysis was based on the uniaxial tensile testing data of dog-bone NinjaFlex^®^ specimens with wall lines that composed approximately 56% of the specimen width [12]. A hyperelastic 3rd order Mooney–Rivlin incompressible material model presented the best fit for these data (Figure 4). This model is represented by the following equation [21,22]:

(1) $$\omega =C_{10}\left(I_{1}-3\right)+C_{01}\left(I_{2}-3\right)+C_{11}\left(I_{1}-3\right)\left(I_{2}-3\right)$$

where ω is the strain energy density function and $I_{1}$ and $I_{2}$ represent the first and second invariants of the deformation tensor, expressed by,

(2) $$I_{1}=\lambda_{1}^{2}+\lambda_{2}^{2}+\lambda_{3}^{2}$$

(3) $$I_{2}=\lambda_{1}^{2}\lambda_{2}^{2}+\lambda_{2}^{2}\lambda_{3}^{2}+\lambda_{3}^{2}\lambda_{1}^{2}$$

Lastly, $\lambda_{i}$ represents the stretch of the specimen, or λ=1+ε, with ε being the strain. $C_{10},\;C_{01},$ and $C_{11}$ are listed in Table 3.

Based on the stress strain curve of Figure 4, *E* was assigned a value of 9.45 MPa by calculating the slope of the linear range of the stress–strain curve: 0 to 0.2 m/m [12]. This value is lower than that reported by the manufacturer, but falls within the range reported by other researchers, between 5.24–12.2 MPa [8,9,11,12,15] (Table 1).

### 2.4. Analytical Modeling Using Modified Euler–Bernoulli Equations

Classical equations of material mechanics can be used to predict the component deformations under known loads. For example, traditional beam theory was used to model a multibody system composed of NinjaFlex^®^ and rigid links of acrylonitrile butadiene styrene (ABS) [23]. Similarly, Euler–Bernoulli beam theory was used to model hyperelastic pneumatic actuators made of silicone [3]. However, classical beam equations may not be able to accurately describe the deformation of parts such as those made of NinjaFlex^®^ due to the hyperelastic material behavior and the large deflection these parts may experience.

**Figure 5:**
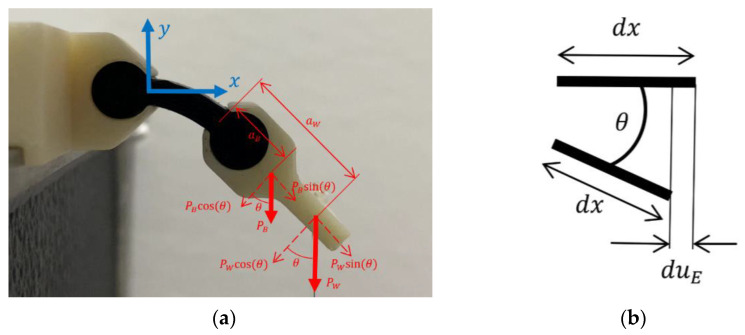
Specimen diagrams. (**a**) Diagram of experimental system: $P_{w}$ is the load applied, $P_{B}$ is the load applied by the weight of the bracket. The lengths $a_{B}$ and $a_{W}$ represent the moment arms for each respective load. The angle θ is equivalent to $\tan^{-1}\left(v_{x}\left(l\right)\right)$ at the tip of the beam. (**b**) Retraction of a differential element on inextensible beam.

In this section, the use of a linear material model for the MEB method was deemed appropriate if the maximum strain is with the linear portion of Figure 4. We propose modifying the Euler–Bernoulli beam theory to account for the deformation of the beam in the axial direction due to bending. As such, the component of the normal load will be a function of the load point’s rotation and the load variation (Figure 5a). This accounted for the forces and moments acting upon the experimental specimen as well as the load variation as the angle of deflection increased. Euler–Bernoulli beam equations were used as a basis, though through the comparison of the virtual work done on the system and the strain energy in the system, the deflections of the beams were derived. Therefore, the virtual work and virtual strain energy of the system were assumed to be equivalent.

It was assumed that the cylindrical ends were fitted perfectly within the fixtures with no slippage during the experiment (Figure 5a). The free-end bracket had a significantly higher stiffness than the specimen and was assumed to be rigid. To analyze the deflection of the specimens, the variations in the virtual work and strain energies were equated to zero:

(4) $$\delta W-\delta U_{A}-\delta U_{B}=0$$

where *W* is the virtual work, *U_A_* is the axial strain energy, and *U_B_* is the bending strain energy. The work is defined as:

(5) $$\begin{array}{c} W=\left(P_{W}+P_{B}\right)sin\left(\theta \left(l\right)\right)u\left(l\right)+\\ \left(P_{W}+P_{B}\right)\cos\left(\theta \left(l\right)\right)v\left(l\right)+\\ \left(P_{W}a_{W}+P_{B}a_{B}\right)\cos\left(\theta \left(l\right)\right)\theta \left(l\right) \end{array}$$

where $P_{W}$ is the load from the applied weight, $P_{B}$ is the load from the bracket weight, *θ* is the angle between the plane cross-section and the vertical axis, *u* is displacement in the *x*-direction, *v* is displacement in the *y*-direction, $a_{W}$ is the moment arm from the applied weight, and $a_{B}$ is the moment arm from the weight of the bracket.

Or simplifying,

(6) $$W=P\left(sin\left(\theta \left(l\right)\right)u\left(l\right)+\cos\left(\theta \left(l\right)\right)v\left(l\right)\right)+M\cos\left(\theta \left(l\right)\right)\theta \left(l\right)$$

where the total applied load is $P=P_{W}+P_{B}$ and the total applied moment is $M=P_{W}a_{W}+P_{B}a_{B}$.

It should be noted that the slope of the tip point is related to the deflection derivative using the following equation:

(7) $$\theta \left(l\right)=\tan^{-1}\left(v_{x}\left(l\right)\right)$$

The strain energy equations for both the axial and bending strain are as follows:

(8) $$U_{A}=\frac{1}{2}EA\int_{0}^{l}u_{x}^{2}dx$$

(9) $$U_{B}=\frac{1}{2}EI\int_{0}^{l}v_{xx}^{2}dx$$

where *E* is the modulus of elasticity, *A* is the cross-sectional area, and *I* is the area moment of inertia of the rectangular cross-section. *A* and *I* are defined as:

(10) A=wh

(11) $$I=\frac{1}{12}wh^{3}$$

Equations (8) and (9) lead to these two differential equations in terms of *v* and *u*:

(12) $$v_{xxxx}=0$$

(13) $$u_{xx}=0$$

These differential equations are subject to the following displacement boundary conditions:

(14) $$v\left(0\right)=v_{x}\left(0\right)=u\left(0\right)=0$$

Additionally, these differential equations have the following force boundary conditions:

(15) $$Pcos\left(\theta \left(l\right)\right)=-EIv_{xxx}\left(l\right)$$

(16) $$-Psin\left(\theta \left(l\right)\right)\left(\frac{1}{1+v_{x}^{2}\left(l\right)}\right)v\left(l\right)-Msin\left(\theta \left(l\right)\right)\left(\frac{1}{1+v_{x}^{2}\left(l\right)}\right)\theta \left(l\right)\;+Mcos\left(\theta \left(l\right)\right)\left(\frac{1}{1+v_{x}^{2}\left(l\right)}\right)+Pcos\left(\theta \left(l\right)\right)\left(\frac{1}{1+v_{x}^{2}\left(l\right)}\right)u\left(l\right)=EIv_{xx}\left(l\right)$$

or,

(17) $$\left(\frac{1}{1+v_{x}^{2}\left(l\right)}\right)\left(\left(Pu\left(l\right)+M\right)\cos\left(\theta \left(l\right)\right)-\left(Pv\left(l\right)+M\theta \left(l\right)\right)\sin\left(\theta \left(l\right)\right)\right)=EIv_{xx}\left(l\right)$$

(18) $$Psin\left(\theta \left(l\right)\right)=EAu_{x}\left(l\right)$$

The solutions to the deflections *u* and *v* are:

(19) $$u\left(x\right)=Dx$$

(20) $$v\left(x\right)=\frac{1}{6}Bx^{3}+\frac{1}{2}Cx^{2}$$

Using the force boundary conditions,

(21) $$B=-\frac{P}{EI}\cos\left(\theta \left(l\right)\right)$$

(22) $$C=\frac{\left(\left(Pu\left(l\right)+M\right)\cos\left(\theta \left(l\right)\right)-\left(Pv\left(l\right)+M\theta \left(l\right)\right)\sin\left(\theta \left(l\right)\right)\right)+PL\cos\left(\theta \left(l\right)\right)}{EI\left(1+v_{x}^{2}\left(l\right)\right)}$$

(23) $$D=\frac{P}{\mathrm{EA}}\sin\left(\theta \left(l\right)\right)$$

It can be seen that *B*, *C*, and *D* are functions of the external forces, specimen dimensions, and $u\left(x\right)$, $v\left(x\right)$, and $v_{x}\left(x\right).$ These three equations can be solved simultaneously using the MATLAB^®^ function *fsolve*, which requires an initial guess. It was decided to use the classical beam theory to provide the initial guess for the first step, i.e., a beam is deforming under the weight of the bracket. In this case the initial guesses for *B*, *C*, and *D* were $-\frac{P_{B}}{EI},\;\frac{P_{B}l+P_{B}a_{B}}{EI},\;\mathrm{and}\;\frac{P_{B}l}{EA}$ respectively. Subsequently, the results of an iteration were used as the initial guesses for the next one. To ensure their convergence of the solution, each load step was divided into ten sub-steps.

The deflection equations of the previous section only described bending and axial deflection. These equations, however, do not account for the retraction along the *x* direction that is typically associated with large deformation [24]. Based on Figure 5b, the differential retraction, $du_{E}$, of the beam can be expressed as (24).

(24) $$du_{E}=dx\left(1-\cos\left(\theta \right)\right)=dx\left(2sin^{2}\left(\frac{\theta }{2}\right)\right)$$

Integrating this equation, the retraction of the tip point, $u_{E}$, can be obtained:

(25) $$u_{E}=-\int_{0}^{l}\left(2sin^{2}\left(\frac{\tan^{-1}\left(v_{x}\right)}{2}\right)\right)\;dx$$

Applying the derivative of Equation (20), the tip point retraction equation becomes:

(26) $$u_{E}=-\int_{0}^{l}\left(2sin^{2}\left(\frac{\tan^{-1}\left(\frac{1}{2}Bx^{2}+Cx\right)}{2}\right)\right)\;dx$$

The total deformation of the tip point can be expressed as a combination of the retraction in addition to the extension caused by $\left(P_{W}+P_{B}\right)sin\left(\theta \left(l\right)\right)$:

(27) $$u\left(l\right)=-\int_{0}^{l}\left(2sin^{2}\left(\frac{\tan^{-1}\left(\frac{1}{2}Bx^{2}+Cx\right)}{2}\right)\right)\;dx+Dl$$

(28) $$v\left(l\right)=\frac{1}{6}Bl^{3}+\frac{1}{2}Cl^{2}$$

### 2.5. Finite Element Analysis Modeling

Several researchers investigated the factors that affect the modeling of 3D printed components using FEA [25,26]. While these studies were all reasonably successful, showing the desired material parameters or predictive capabilities, they lacked focus on hyperelastic materials such as NinjaFlex^®^ as well as large deflection. By understanding the bending behavior of 3D printed hyperelastic polymers, they may be used in a wider range of applications.

**Figure 6:**
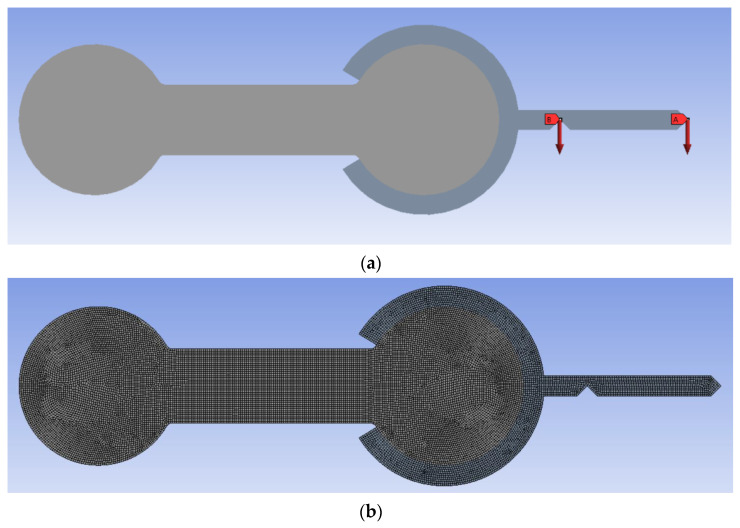
FEA model: (**a**) forces applied to the FEA model representing the weight of the bracket and the weights; (**b**) a typical mesh for an average element size of 0.10 mm.

ANSYS^®^ (simulation software, ANSYS, Inc., Canonsburg, PA, USA) was used for the FEA simulations [27]. The model of the specimen was imported as a 2D IGES file and processed as a plane stress problem The free end bracket was simplified from the one used in the experiments (Figure 2) to reduce the computational load of the simulations. To ensure that this model was comparable to the experiment, three issues were addressed. Firstly, since the bracket was significantly stiffer than the specimens, it was assigned structural steel material properties. Secondly, the center of gravity’s location was maintained. Lastly, gravity was turned off, and the weight of the actual fixture was applied as a force in the center of gravity’s location. Figure 6a shows the model. The values of said weights are found in Table A2. A mesh stability study was conducted, and it was found that an average element size of 0.10 mm was stable. The number of elements in simulations ranged from 5483 elements in Specimens 1–3 to 17,999 in Specimens 16–18. Figure 6b represents a typical mesh.

## 3. Results and Discussion

The maximum strain calculated by FEA for any model did not exceed 0.16 mm/mm, which is within the linear range of Figure 4. Therefore, the modulus of elasticity, *E*, in the modified Euler–Bernoulli model was assigned a value of 9.45 MPa, as shown in Section 2.2.

**Figure 7:**
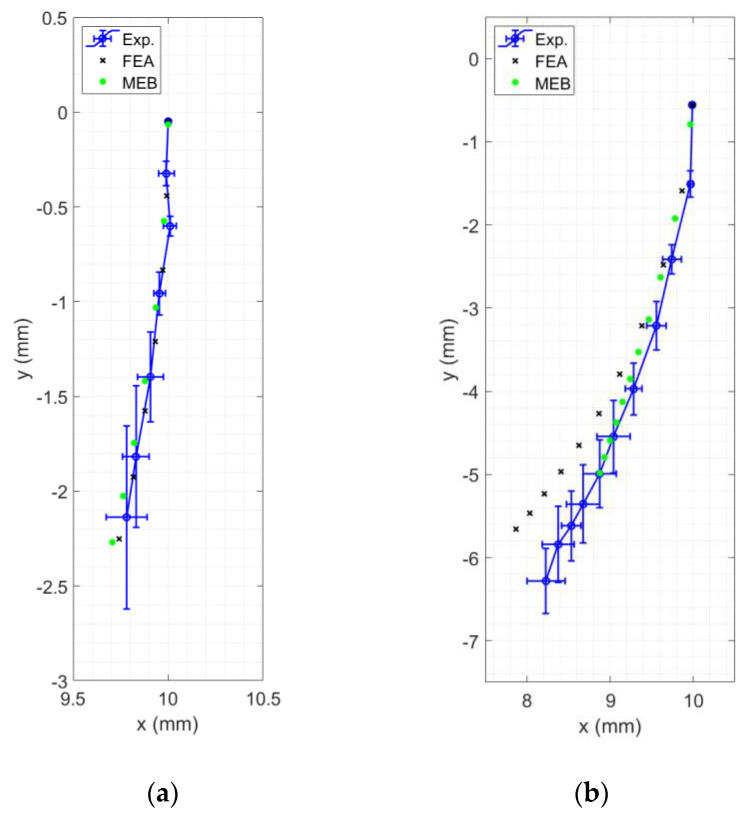
Comparison of experimental, FEA, and MEB deflections: (**a**) a relatively stiff Specimen 15; (**b**) a relatively flexible Specimen 1.

The experimental results for the three samples of each specimen were averaged, and their standard deviations were calculated in the vertical and horizontal, y and x, directions. The corresponding MEB and FEA results were also recorded. Figure 7 shows the resulting deflections of one of the stiffest and most flexible specimens, Specimen 15 and 1, respectively. The dimensions of the specimens are listed in Table A1.

In the case of Specimen 15, which was relatively stiff, the experimental tip deflection deviated slightly from moving along a smooth curve, most noticeably in the *x*-direction. On the other hand, the tip point of Specimen 1 moved along a smoother curvature. All other tested specimens followed these trends. For example, the tip points of Specimens 7–9 and 13–15, which were relatively stiff, did not follow a smooth curvature. This may be the combined result of small magnitudes of motion, the camera quality, and the inevitable blur. Additionally, it was observed that the standard deviation increased as loading increased for all specimens, which may be the result of built-up issues with the experimental setup or variation in the 3D printer quality.

The MEB method reasonably predicted the deflection of the relatively stiff Specimen 15. The deflection was overestimated for the lower loads and then underestimated as the load increased. For Specimen 1, the MEB lagged behind the experimental results, although it followed the average experimental tip point curvature. The MEB method worked generally well for stiff specimens, such as Specimen 15. For all tested specimens, the curvature of the MEB deflection consistently followed the curvature of the experiments.

The FEA method slightly overestimated the deflection of Specimen 15. Similarly to MEB, the curvature of the FEA tip point defection matched the experiment well. However, the FEA resulted in an overestimation of the tip point retraction in the case of Specimen 1, as shown in Figure 7b. A possible reason for this behavior may be due to the way ANSYS analyzes bodies experiencing large deflection. When a body experiences large strain, the deformation triggers reorientation of the applied loads. It may be possible that this load distribution was less accurate, resulting in the excessive retraction that can be observed in the figure.

To compare the experimental and modeling data, the deflection under the weight of the bracket alone, $P_{B}$, was considered the datum. A measure of the difference of each loading case of a specimen was performed. These measures were averaged as follows:

(29) $${Ζ}_{m,i}=\frac{\sum_{j=1}^{J_{i}}\frac{\sqrt{{\left(xe_{i,j}-x_{m,i,j}\right)}^{2}+{\left(ye_{i,j}-y_{m,i,j}\right)}^{2}}}{l_{i}+a_{w}{}_{i}}}{J_{i}}$$

where *m* is the modeling method (MEB or FEA), *I* is the specimen number, *j* is the load number, $J_{i}$ is total number of loads, xe, $x_{m}$,  ye and $y_{m}$ are the experiment and model deflection in the *x* and *y* directions, respectively, and $l_{i}$ is the length of the specimen.

All results from experiments, MEB, and FEA were combined using the following nondimensional stiffness parameter, *Q* [28,29,30]:

(30) $$Q_{i}=\frac{\left(P_{B}{}_{i}+\max\;(P_{W}{}_{i})\right){\left(l_{i}+a_{W}{}_{i}\right)}^{2}}{EI_{i}}$$

It should be noted that lower *Q* values typically correspond to higher bending stiffness.

**Figure 8:**
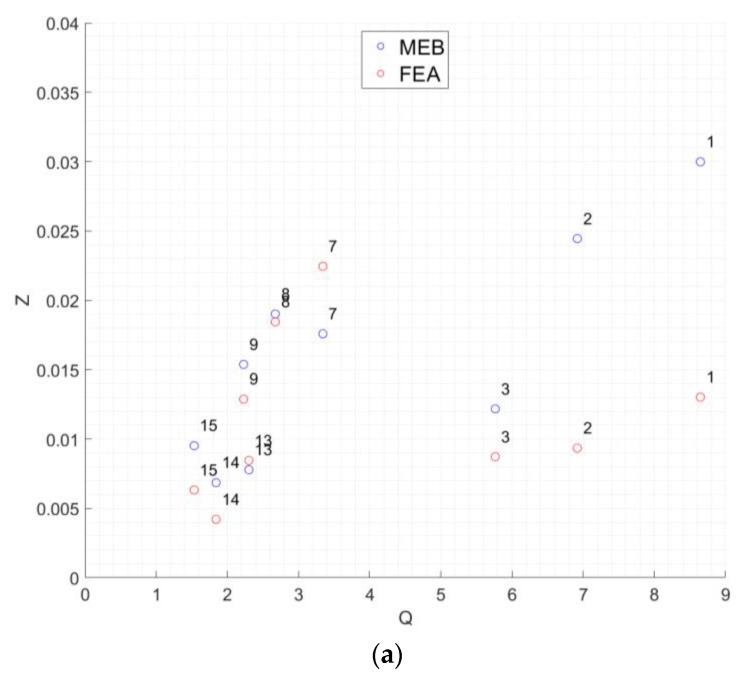

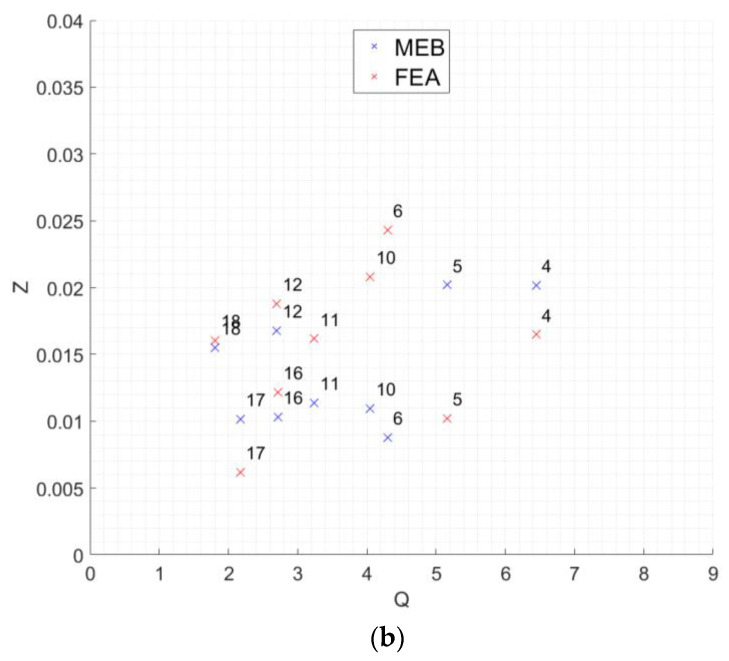
Deviation of MEB and FEA with respect to average experimental results; (**a**) specimens with l=10 mm; (**b**) specimens with l=15 mm.

Overall, MEB underestimated the deflection of most specimens when compared to the respective experimental results, while FEA overestimated them. The results of the average difference,$\;{Ζ}_{m}$, were plotted with respect to the non-dimensional parameter *Q* (Figure 8). The data were split by length for clarity. For specimens with l=10 mm length (Figure 8a), both MEB and FEA predictions were of the same order for values of Q below 4. The same observation was valid for all tested specimens in the case of l=15 mm (Figure 8b). However, when l was equal to 10 mm and Q was above 5, the FEA was more accurate. However, the retraction along the *x* direction was significantly higher than that with MEB, where the displacement closely followed the experimental curve, as can be seen in Figure 8b. It was also observed that the FEA showed more stable *Z* values as *Q* increased.

## 4. Conclusions

This research aimed to characterize the bending behavior of 3D printed hyperelastic polymer components and to develop a relatively simple model that can predict the deformation of these components accurately. A series of bending experiments were conducted on 18 different cantilever specimens with significantly different flexibilities. A model was developed based on fundamental beam theory: the modified Euler–Bernoulli (MEB) model, to account for the large deformations and planar motion of the specimens. A finite element analysis was also conducted to predict the bending behavior. The results of this MEB model and FEA were compared to the experimental data, indicating that both approaches produced accurate results for most specimens. However, the MEB underestimated the overall deflection, while the FEA resulted in a significantly larger retraction of the specimens under load.

MEB can sufficiently provide an accurate prediction of deformation for a large range of hyperelastic 3D printed polymer specimens and has several advantages over FEA, including simplicity and speed. MEB exhibited consistently more accurate retraction prediction, with the deflection of the tip point aligning with the experimental results; however, the deflection in the y-direction was underestimated. The cause of this may be due to the fundamental theory used to derive the MEB method, the perfectly linear material model, or the assumption of a homogeneous isotropic specimen. FEA predicted more accurate deflection in the y-direction and maintained relatively accurate retraction under most circumstances. However, the highly flexible specimens modeled by FEA exhibited overestimation of retraction. This may be due to the print geometry within the specimen. As shown in Table 1, the mechanical characteristics of the material used exhibited variability caused by differences in the type of 3D printer, printer settings, and filament size, including characteristics such as modulus of elasticity, yield strength, ultimate strength, and hardness. For a more complete understanding, numerous polymers with a wide range of mechanical characteristics should be tested and modeled using the methods described. This way, the gradient of results would show a clearer behavior of hyperelastic polymers. Alternatively, the excessive retraction in the flexible specimens exhibited in the FEA results may be mitigated by different material models that represent the behavior better. Additionally, the mechanical properties of the core portion, which is printed using 45° lines, are expected to differ from those of the wall lines. These variations pose a challenge to modeling components made of 3D printed polymers, especially those undergoing large deflection. An in-depth study of these parameters should be considered for future work.

## Figures and Tables

**Table 1:** NinjaFlex mechanical characteristics.

Source	Yield Strength, (MPa)	Ultimate Strength, (MPa)	Tensile Modulus, (MPa)	Elongation at Yield, (%)	Elongation at Break, (%)	Toughness, (m × N/m^3^ × 10^6^)	Hardness, (Shore)
Manufacturer [15]	4.00	26.00	12.00	65.00	660	82.70	85A
Pitaru et al. [8]	2.80	-	8.51	51.85	-	-	-
Mogan et al. [9]	-	13.19	-	-	-	-	55.7D
Holmes et al. [10] ^1^	0.16	-	1.50	-	-	-	-
Messimer et al. [11], 25% infill	-	7.01	5.17	-	476	-	-
Messimer et al. [11], 50% infill	-	8.61	5.19	-	487	-	-
Messimer et al. [11], 75% infill	-	10.21	5.22	-	497	-	-
Messimer et al. [11], 100% infill	-	11.81	5.24	-	508	-	-
Reppel and Weinberg [12]	-	27.80	12.2	-	1200	133.40	-

^1^ The values reported are for gyroid printed samples.

**Table 2:** Print parameters.

Print Parameter	Value
Filament Diameter	1.75 mm
Infill Density	100%
Quality	Standard (0.32 mm layer height)
Nozzle Temperature	225 °C
Bed Temperature	60 °C
Print Speed	15 mm/s

**Table 3:** Model parameters for the 3^rd^ order Mooney–Rivlin model used in the FEA model.

C10	C01	C11	D1
−80,337	2,348,300	30,693	0

## Data Availability

The data presented in this study are available on request from the corresponding author.

## Appendix A

**Table A1:** Specimen dimensions.

Specimen ID	*l* (mm)	*h* (mm)	*w* (mm)	*d* (mm)	Number of Wall Lines
1	10.0	1.8	8	3.85	2
2			10	3.85	2
3			12	3.85	2
4	15.0	1.8	8	3.85	2
5			10	3.85	2
6			12	3.85	2
7	10.0	2.7	8	5.70	3
8			10	5.70	3
9			12	5.70	3
10	15.0	2.7	8	5.70	3
11			10	5.70	3
12			12	5.70	3
13	10.0	3.6	8	7.70	3
14			10	7.70	3
15			12	7.70	3
16	15.0	3.6	8	7.70	3
17			10	7.70	3
18			12	7.70	3

**Table A2:** Specimen loading configurations.

Specimen ID	Load Increment (g)	Maximum Applied Load (g)	Bracket Weight (g)	Center of Mass of the Right Bracket (mm) ^1^	Location of External Weights ^1^ (mm)
1, 2, 3	5	50	3.88	9.3	17.86
4, 5, 6	5	25	3.88	9.3	17.86
7, 8, 9	10	70	3.70	9.5	17.21
10, 11, 12	10	60	3.70	9.5	17.21
13, 14, 15	20	120	3.41	10.4	16.87
16, 17, 18	20	100	3.41	10.4	16.87

^1^ Distances are measured from the left edge of the right cylindrical end of the specimen, $a_{W}$ in Figure 4.
